# A case report of male breast cancer treated with breast-conserving surgery

**DOI:** 10.1016/j.ijscr.2025.111804

**Published:** 2025-08-19

**Authors:** Kimiyasu Yoneyama, Motohito Nakagawa, Asuka Hara

**Affiliations:** Department of Breast Surgery, Hiratsuka City Hospital, Hiratsuka, Japan

**Keywords:** Male breast cancer, Breast-conserving surgery, Mastectomy, Cosmetic, Case report

## Abstract

**Introduction and importance:**

Male breast cancer is a rare disease that accounts for less than 1 % of all breast cancer cases. The most common surgical treatment for male breast cancer is mastectomy, but breast-conserving surgery has been performed increasingly in recent years. This report describes a case of male breast cancer that was treated by breast-conserving surgery.

**Case presentation:**

A 61-year-old man visited our hospital complaining of a painless mass in his left breast. Imaging revealed a well-defined cystic mass, aspiration of which revealed bloody fluid. Cytology was inconclusive, but the findings were suggestive of malignancy. A left lumpectomy was performed for diagnostic purposes. Postoperative pathology revealed invasive ductal carcinoma with a predominant intraductal component. The surgical margins were negative. The patient declined adjuvant hormone therapy and radiotherapy and has been kept under follow-up in the year since surgery. There has been no recurrence during this time.

**Clinical discussion:**

As in women, the goals of treatment for breast cancer in men are oncological safety and satisfactory cosmetic results. In the absence of specific guidelines for the treatment of male breast cancer, mastectomy is currently the standard treatment. However, in recent years, breast-conserving surgery has been performed for tumors that are discovered early and do not invade the nipple and areola. If curability and safety can be guaranteed, breast-conserving surgery can be considered for male breast cancer.

**Conclusion:**

Breast-conserving surgery can be an effective treatment option in men with breast cancer.

## Highlights

• Male breast cancer is very rare and is reported to be a source of stigma.
• Although breast-conserving surgery has had good outcomes, men are more likely to undergo mastectomy.
• This case demonstrates that breast-conserving surgery can effectively treat male breast cancer while achieving favorable cosmetic results.

## 1 Introduction

Male breast cancer (MBC) is rare, accounting for less than 1 % of all breast cancer cases and less than 1 % of all cancers in men [[1], [2], [3], [4]]. The median age at diagnosis is slightly older in men (67 years) than in women (62 years) [5]. MBC typically presents as a unilateral, painless, palpable mass in the central subareolar area or slightly distal to the nipple, with nipple invasion at a relatively early stage. Most MBCs are invasive ductal carcinomas. As with postmenopausal breast cancer, approximately 65 %–90 % of MBCs are estrogen receptor- and progesterone receptor-positive. Several studies have shown that the mortality rate is higher for MBC than for breast cancer in women [6], which is attributed to the advanced stage of the disease and the older age of patients at diagnosis [7,8]. Surgical treatment for MBC generally involves mastectomy [[9], [10], [11]], with one report indicating that 71 % of these patients undergo mastectomy [10]. However, in recent years, minimally invasive surgery has been increasingly performed for MBC, as for female breast cancer [12]. However, in a study by Zaenger et al., 56 % of patients with MBC had stage T1 disease, but only 4 % underwent breast-conserving surgery [13]. Therefore, breast-conserving surgery cannot be considered common in MBC. This report describes our experience with a case of MBC in which breast-conserving surgery was performed. The manuscript was written in accordance with the SCARE criteria [14].

## 2 Case presentation

A 61-year-old man was admitted to our hospital complaining of a painless mass in his left breast, which he had noticed 1 month earlier. There was no family history of breast or ovarian cancer.

**Fig. 1:**
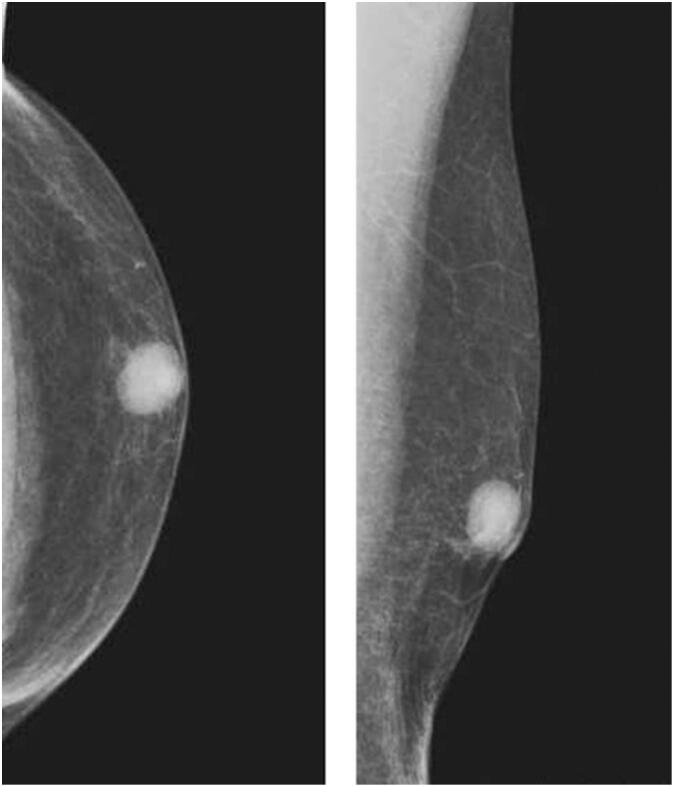
A highly dense mass measuring 1.8 cm in diameter with a clear and regular border was found directly below the left nipple.

**Fig. 2:**
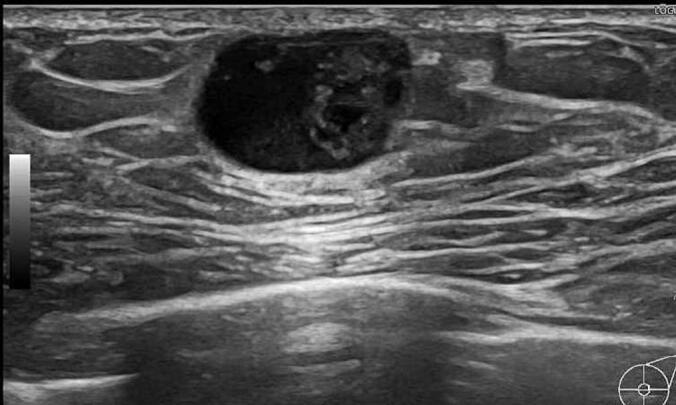
A cystic mass with a clear and regular border and containing liquid components was found.

**Fig. 3:**
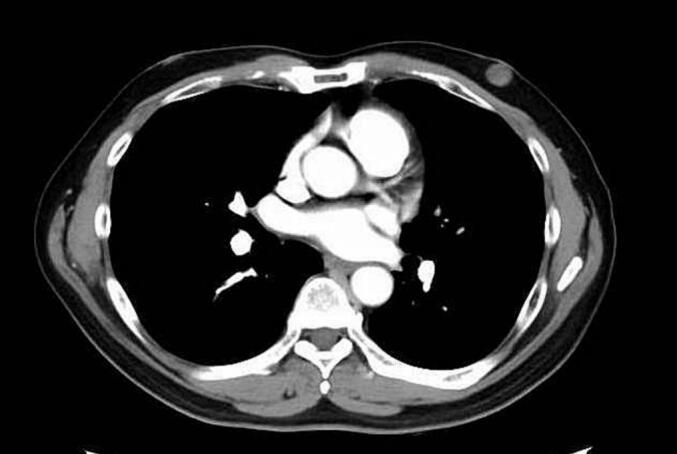
A contrast-enhanced computed tomography scan revealed a mass with a clear border directly below the nipple.

Palpation revealed a clearly demarcated mass measuring 1.5 cm in diameter just below the left nipple. Mammography revealed a dense 1.8-cm mass with a clear border and regular margins just below the nipple of the left breast (Fig. 1). Ultrasound examination showed a cystic mass with a clear border, regular margins, and a liquid component (Fig. 2). There was no evidence of intraductal extension. Contrast-enhanced CT revealed a clearly demarcated mass just below the nipple (Fig. 3). No lymph node enlargement was observed on imaging.

**Fig. 4:**
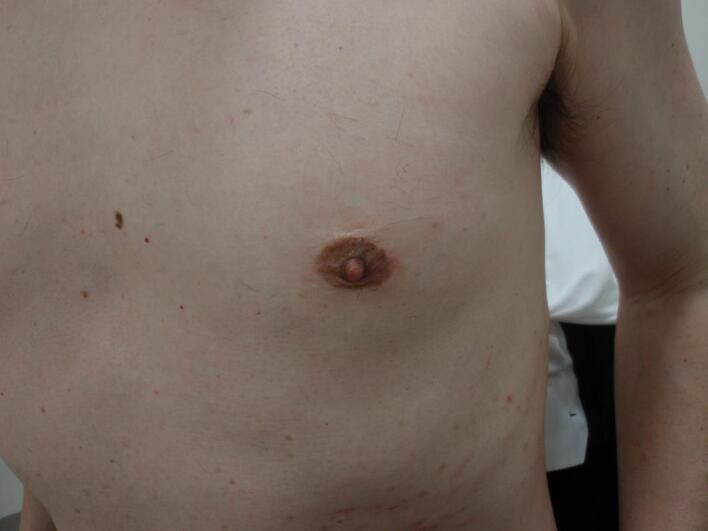
The postoperative scar was not noticeable, and the local cosmetic outcome was good.

The mass was punctured, and bloody fluid was aspirated. Cytology of the internal fluid did not provide a definitive diagnosis of malignancy, but malignancy was strongly suspected based on the imaging findings. Therefore, a lumpectomy was performed. The surgery was performed via a periareolar incision, with a wide resection margin to avoid damaging the capsule of the cyst. The local cosmetic outcome was good and considered satisfactory by the patient (Fig. 4).

**Fig. 5:**
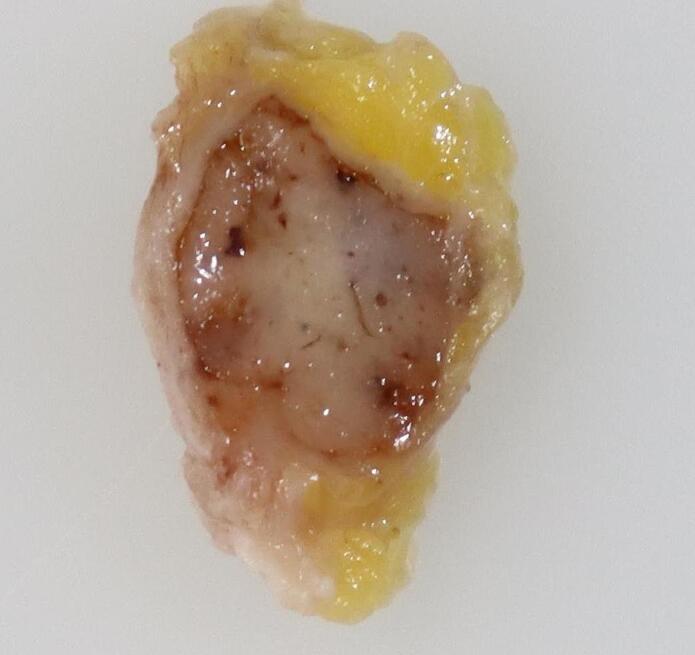
Macroscopically, the tumor was white and cystic with clearly defined borders.

**Fig. 6:**
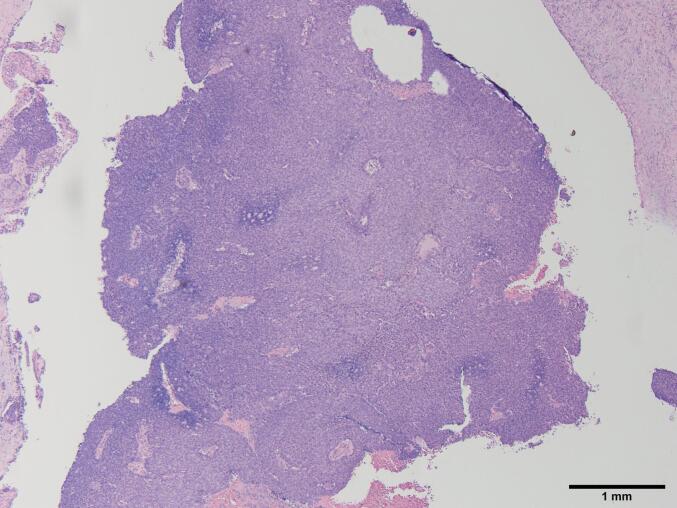
Atypical cells were proliferating as solid components within cystic structure, with slight infiltration into the surrounding stroma. The diagnosis was invasive ductal carcinoma with predominantly intraductal components.

To the naked eye, the tumor appeared as a white cystic lesion with a clear border (Fig. 5). Histopathological examination revealed a proliferation of atypical cells proliferated as solid components within the cystic structure, with slight infiltration into the surrounding stroma. The tumor was diagnosed as invasive ductal carcinoma with a predominant intraductal component (Fig. 6). The resection margin was negative. Immunohistochemical staining showed the tumor to be estrogen receptor-positive, progesterone receptor-positive, and HER2-negative with a Ki-67 of 10 %.

In view of the negative resection margin, we decided not to perform additional resection, and based on the pathological findings, we did not perform a sentinel lymph node biopsy. We offered hormone therapy and breast radiation, but the patient refused. Therefore, we could only observe his condition after surgery. The observation period is still short, one year after surgery, but there has been no recurrence, including locally. A BRCA test performed at a later date was negative.

## 3 Discussion

MBC is very rare, accounting for less than 1 % of all breast cancers. The age at which MBC occurs is older than in women, most often in the late 60s, and the majority of cases are histologically diagnosed as invasive ductal carcinoma. MBC is also characterized by a high rate of hormone receptor positivity.

Factors that increase the risk of developing MBC include Klinefelter syndrome, obesity, radiotherapy, and BRCA gene mutations. Only a small number of MBCs are associated with gynecomastia, and it is not certain whether gynecomastia increases the risk of MBC. The tumor is most often located just below the nipple, which is thought to be because there is only a small amount of ductal tissue just below the nipple in men.

MBC is reported to be found at a more advanced stage than female breast cancer [15,16]. However, MBC is not more biologically malignant than breast cancer in women [17]. Historically, most studies have found that the prognosis of MBC is poor [[18], [19], [20]]. However, there has been a report comparing the survival rates of male and female patients with breast cancer who were matched for disease stage and age at diagnosis [16]. The 10-year survival rates were similar in both sexes. The report concluded that the prognosis is the same for male and female patients with breast cancer of the same disease stage.

Approximately 15 %–20 % of patients with MBC have a family history of breast cancer [21]. In men, BRCA1 does not appear to be associated with a significantly increased risk of breast cancer, although mutations in the BRCA1 gene have been reported in men with the disease [22]. However, men with BRCA2 mutations are more likely to develop breast cancer.

The rate of hormone receptor positivity is higher in MBC than in female breast cancer. One study found that 81 % of MBCs were estrogen receptor-positive and 74 % were progesterone receptor-positive [21].

Breast-conserving therapy is widely used in female breast cancer, but lumpectomy is generally considered contraindicated in men because of the small volume of breast tissue [[23], [24], [25]]. However, many men who develop breast cancer have some degree of breast enlargement, and lumpectomy may be possible. Breast conservation is possible in appropriately selected patients. Men without obvious involvement of the nipple and areola can safely undergo wide excision and radiotherapy with reasonable local recurrence rates and acceptable cosmetic results [10]. However, it is impossible to confirm this because of the small number of cases of MBC.

Preservation of the nipple–areola complex in male patients treated for breast cancer may have a psychological effect as favorable as that in female patients. In our case, the patient was very satisfied with the cosmetic outcome. Breast-conserving therapy should be considered for male patients if possible unless there is obvious invasion of the nipple or areola. A focus on cosmetic appearance is also important in MBC.

Despite the proven efficacy of breast-conserving surgery in women, men are still mostly treated by mastectomy [26]. One study reported that the prognosis was better in patients who underwent breast-conserving surgery than in those who underwent total mastectomy [27]. Another study found no difference in oncological outcome or overall survival between patients who underwent mastectomy and those who underwent breast-conserving surgery [28].

There is a general perception that breast cancer is a woman's disease, and prejudice against men with breast cancer has been reported, with psychological and social consequences for men with the disease [26,29,30].

The change in body image that comes with surgery is equally important for men and women, indicating a need to consider breast-conserving surgery when possible for both sexes [26].

The idea that removal of a breast is not as traumatic for a man as it is for a woman is no longer tenable. Therefore, mastectomy should not be chosen in men or women without careful consideration. Of course, mastectomy will generally be the most common option. However, if the disease is detected early and there is no involvement of the nipple or areola, breast-conserving surgery may be offered to the patient, if possible.

## 4 Conclusion

We have performed breast-conserving surgery in a case of MBC. In cases of MBC without nipple-areola involvement, breast-conserving surgery may be offered if possible.

## Author contribution

**Kimiyasu Yoneyama**: Conceptualization, Investigation, Resources, Writing – Original draft preparation, Writing – Review and editing

**Asuka Hara**: Conceptualization, Investigation

**Motohito Nakagawa**: Administration, Review

## Consent

Written informed consent was obtained from the patient for publication of this case report and accompanying images.

## Ethical approval

Because this paper is a case report, ethical approval from Hiratsuka City Hospital is not required.

## Research registration number

This is a case report, and no registration was required.

## Provenance and peer review

Not commissioned, externally peer-reviewed.

## Funding

This research received no specific grant from any funding agency in the public, commercial, or not-for-profit sectors.

## Conflict of interest statement

None.
